# Validation of the adaptive scan method in the quest for time-efficient methods of testing auditory processes

**DOI:** 10.3758/s13414-023-02743-z

**Published:** 2023-06-22

**Authors:** E. S. Lelo de Larrea-Mancera, T. Stavropoulos, A. A. Carrillo, K. N. Menon, E. C. Hoover, D. A. Eddins, F. J. Gallun, A. R. Seitz

**Affiliations:** 1 Northeastern University, College of Science, Psychology; Center for Cognitive and Brain Health, Boston, MA USA; 2 University of California Riverside, UCR Brain Game Center for Mental Fitness and Wellbeing, Riverside, CA USA; 3 Department of Hearing and Speech Sciences, University of Maryland at College Park, College Park, MD USA; 4 University of South Florida, Communication Sciences and Disorders, Tampa, FL USA; 5 Oregon Health & Science University, Oregon Hearing Research Center, Portland, OR USA

**Keywords:** Psychoacoustics, Psychometrics/testing, Physiological psychology

## Abstract

A major barrier to the clinical application of psychophysical testing of central auditory processes is the time required to obtain precise estimates of different listening abilities. In this study, we validate a novel adaptive scan (AS) method of threshold estimation that is designed to adapt on a range of values around threshold rather than on a single threshold value. This method has the advantage of providing the listener with greater familiarity with the stimulus characteristics near threshold while maintaining precise measurement and increasing time-efficiency. Additionally, we explore the time-efficiency of AS through comparison with two more conventional adaptive algorithms and the method of constant stimuli in two common psychophysical tasks: the detection of a gap in noise and the detection of a tone in noise. Seventy undergraduates without hearing complaints were tested using all four methods. The AS method provided similar threshold estimates with similar precision to those from the other adaptive methods and, thus, it is a valid adaptive method of psychophysical testing. We also provide an analysis of the AS method based on precision metrics to propose a shortened version of the algorithm that maximizes the time/precision tradeoff and can achieve similar thresholds to the adaptive methods tested in the validation. This work lays the foundation for using AS across a wide variety of psychophysical assessments and experimental situations where different levels of precision and/or time-efficiency may be required.

**Supplementary Information:**

The online version contains supplementary material available at 10.3758/s13414-023-02743-z.

## Introduction

Several psychophysical tests that could convey useful information about auditory processes are relatively absent in clinical practice in part due to their potential costs. These costs range from those associated with the need for special test facilities, such as a sound booth, or specialized equipment, such as audiometers and high-quality audio speaker systems or setups. There are also costs associated with the time spent by clinicians, who often have very little time per patient, and by auditory researchers, who need to select which tests to run within the time constraints imposed by psychoacoustical testing. Further, the time spent by the patient is a concern, where each test adds to the burden of a given visit, in addition to the costs associated with travelling to and from appointments for the repeated visits that may be necessary to achieve an accurate diagnosis. Given these potential costs, complementary information from psychophysical testing, useful as it might be, will only become feasible for translation to the clinic to the extent that new tools are developed that can mitigate these costs.

Recently, we have developed PART (Portable Automated Rapid Testing), a tablet-based application capable of implementing diverse psychophysical tests with laboratory-grade quality (Gallun et al., 2018). PART has been validated on several psychophysical assessments in young normal-hearing populations with commercially available equipment (under $500), both in isolated laboratory conditions and in more ecologically valid conditions, including headphone variability and environmental noise (Lelo de Larrea-Mancera et al., 2020). Further, results have been replicated for remote testing, participant-owned equipment (Lelo de Larrea-Mancera et al., 2022). The *Portable* and *Automatic* aspects of PART are ideal to address some of the costs of psychophysical testing mentioned above. In this study, we focus on improving the R in PART; that is, we seek to achieve a reliable method for *Rapid* psychophysical testing that can reduce the time-burden of assessing central auditory processes.

We identify two critical factors that impact the number of trials used in psychoacoustical testing. First is the need to familiarize participants naïve to psychophysical testing with the stimuli and tasks in the protocol. Typically, practice runs are delivered to achieve this; but data obtained during practice are usually not considered in the final analyses and can be regarded as “wasted” time. Also, even when practice trials are given, listeners sometimes lose track of the relevant auditory cues needed to perform the task, creating confusion that might conspire against measurement validity. The second factor has to do with the precision of the estimate that is required to draw an adequate conclusion from the data. A hearing screening may require little precision, as simply knowing that a participant can detect a sound at a given intensity (e.g., 20 dB) may be sufficient to conclude that hearing is “normal.” Conversely, an intervention study may require greater precision to characterize perception well enough to observe a modest, but clinically relevant, change in performance. Across different use cases, sometimes highly precise estimates are required, and at other times coarse estimates are sufficient; an efficient estimation method should be flexible to the required precision of the estimate.

To this end, we developed a novel adaptive method we call the adaptive scan (AS), which we here validate and attempt to optimize in terms of time-efficiency. AS adapts on a range of stimulus values rather than a single value. This range – which constitutes a scan – adapts by shifting the range and scaling the distance between steps in a scan. The number of steps in a scan remains constant throughout testing and the stimulus level descends through the range during each scan. Scanning over a range of stimulus levels provides listeners with clear examples of the stimulus characteristics and allows them to hear how the relevant cue changes as the tracked parameter descends to a magnitude where the probability of detection is low. Thus, each scan includes familiarization trials that serve a purpose in threshold estimation rather than being discarded. Additionally, familiarization occurs at different points throughout the adaptive tracking rather than just at the beginning to serve as a repeated reminder of the relevant cues.

Here, we validate AS by comparing the precision of its threshold estimates to ones obtained using the *transformed and weighted up-down* method (T&W) described by García-Pérez (1998) and those obtained from a more classic *transformed* (T) *up-down* method, as described by Wetherill and Levitt (1965). Additionally, we included the method of constant stimulus (CS), classically stipulated by Fechner (1860). The rationale of using the cumbersome CS method was that it is considered a gold-standard in psychophysical testing and can be used to extract the whole psychometric function across the full range of performance. All methods were compared in terms of the estimated threshold, as well as the precision of this estimate.

This study represents a first step in the development of more optimal adaptive methods for portable automatic rapid testing using methods we have previously used (T&W), methods that would be typical (T), and a novel method (AS). Recommendations for an optimal adaptive method and parameters to be used are provided in the context of two classic psychophysical assessments of different domains of hearing and with different expected shapes of the psychometric function: the gap-in-noise (GIN) and the tone-in-noise (TIN) tasks. The results of this study will inform use of AS with other psychophysical tests and will assist the translation of psychophysical work into the clinic in a manner that will reduce the time-burden to researchers, clinicians, and patients.

## Methods

### Participants

Seventy students (*mean age* = 19.42 years, *SD* = 3.5; 45 female) from the University of California, Riverside signed up through a research participation system that provided course credit for their participation. While this was not based upon an explicit power analysis, the number of participants was consistent with the number that we’ve run in prior validation studies with PART with a recruitment plan of targeting 60–80 participants within a period of an academic quarter, with the goal of having 50–60 students that would pass criteria for inclusion in analyses. Data analyses followed full enrollments. All participants self-reported normal hearing and no history of psychiatric or neurological disorders, and provided informed consent approved by the University of California, Riverside Human Research Review Board.

### Outlier rejection

To detect outliers, we applied a measure of threshold spread (Sn) as explained by Jones (2019) and first introduced by Rousseeuw and Croux (1993), and cases with an absolute value beyond Sn*3 were rejected from aggregate analysis. Sn is similar to MAD (median absolute distance), but does not assume symmetric spread of the data as it is based on the median absolute distance of every datapoint from every other datapoint. Participants with four or more outlying threshold values were rejected from all analyses (n = 9). The total number of cases rejected were between 11 and 17 (between 15% and 25%) per method/task combination. All data including outliers are shown in the Online Supplemental Materials (OSM), Figs. S1 and S2.

### Procedures

Two tasks were performed remotely monitored via video call: Gap-in-noise (GIN) detection (e.g., Grose et al., 1989), and Tone-in-noise (TIN) detection (e.g., Wilson et al., 2003). Participants utilized their own uncalibrated devices and were instructed to turn PART’s volume all the way up before starting the experiment. Although the actual output values in different systems would have some level differences, these have not substantially impacted estimated thresholds in our previous work with suprathreshold auditory tests (Lelo de Larrea-Mancera et al., 2020, 2022). PART had an additional mechanism to display a warning for participants if their system was not properly set in its own maximum level. Both tasks consisted of a four-interval, two-cue, two-alternative forced choice where a “standard” stimulus that does not contain the target stimulus (e.g., gap or tone) is presented in the first and fourth intervals as cues (2 cue), and the “target” stimulus is placed in either the second or the third intervals (two alternatives). Participants are forced to choose between the second and third intervals to indicate which interval contained a target. These procedures are the same as those utilized in Lelo de Larrea-Mancera et al. (2020, 2022).

**Fig. 1:**
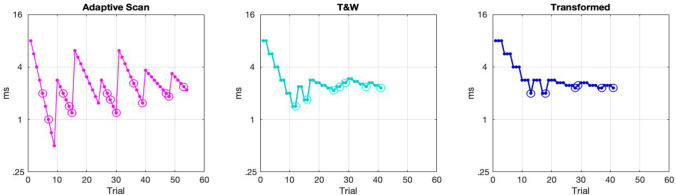
A visual representation of the three adaptive algorithms used: the adaptive scan (AS; left panel), the transformed and weighted method (T&W; center panel), and the transformed method (T; right panel). Correct trials are depicted with a single dot and incorrect trials include an additional circle. As can be seen in the figure, the AS adapts on a range of stimulus values while the T&W and T methods adapt on a single stimulus value

For each task, each participant performed two runs with each of four different psychometric methods (three adaptive and one constant stimulus) across 3 days. The three adaptive tracking methods were: adaptive scan (AS; detailed below), transformed and weighted up-down method (T&W) (e.g., García-Pérez, 1998, 2011), and transformed up-down method (T) (e.g., Levitt, 1971) (see Fig. 1). All adaptive methods were delivered in the first two days of testing. The method of constant stimulus (CS) was presented to participants on the third day. The details of each of these methods is detailed below with the specifics for each task. The design of the experiment was intended so that any noise due to novelty of the instrument would be spread between the adaptive methods tested and the constant stimulus method was delivered in the end to get the closest estimate possible to the true shape of the psychometric function for each participant.

#### Adaptive scan (AS)

The AS consists of a series of progressive tracks or scans (eight in this case), each with a stopping rule of three errors in this case. For the first scan an a priori range of interest was based on previous literature and a small pilot, and is detailed below for each task. Step sizes correspond to nine logarithmic-spaced steps (on a factor of 2) for GIN and nine linearly spaced steps in the case of TIN. After the first scan, the algorithm partitions the scan range into three regions, the first two steps represent a top region, the last two are the bottom, and the remaining (five in this case) are the center region. After the first scan (with nine steps in this case) is complete, AS calculates a region to adapt within that range controlling for guess rate using the formula $(hits- nTrials * guessRate) / (1- guessRate).$ Note that the formula yields the number of steps where the threshold is calculated within a scan. If the calculation of the immediately previous scan leads to the bottom or top regions, AS adaptively slides the range for the next scan up/down by half the scan range width. If the calculation leads to the center region, AS adaptively zooms into this range by dividing the step-size by two and centering the scan range at the calculated region. This way, AS first gets the region of performance where a threshold might be calculated for a given subject in the center region of the scan, and then it zooms into it. A minimum step-size is also set a priori to limit the amount of parametric space zoom allowed. Lastly, the number of steps within a scan remained constant (nine steps in this case) unless three errors within a scan occurred. In this case, the scan was interrupted, and the threshold was calculated in the top region.

### Stimuli

#### Gap in noise

Standard stimuli consisted of white noise of 400-ms duration presented at a nominal level of 70 dB. Target stimuli consisted of a gap placed in the middle of the noise stimulus with a depth of 1 adapting on duration starting from 8 ms (max 60, min 0 ms), while keeping the total duration of the stimulus constant. Adaptive algorithms advanced on an exponential scale divided in 20 linear steps between every major factor of 2 according to their own rules specified below.

- AS method: eight scans, nine steps per scan, three errors to abort a given scan.
- T&W method stage 1: up 15/20 steps, down 10/20 steps; stage 2: up 3/20 steps, down 2/20.
- T method stage 1: 10/20 steps, stage 2: 2/20 steps
- Constant stimuli: nine log steps from 8 to 0.5 ms, eight repetitions.

#### Tone in noise

Standard stimuli consisted of narrow-band noise of 600-Hz bandwidth centered at 500-Hz frequency (linear radial rove of pi*radians), with a linear distribution of 1,000 components. Noise was presented at a fixed nominal level of 70 dB. The signal consisted of a pure tone of 500-Hz frequency (linear radial rove of pi*radians) and 400-ms duration presented simultaneously with the noise. The tone adapted linearly on a level starting from 68 dB (-2 TMR dB) according to the rules of each of the adaptive algorithms specified below.

- AS method: nine windows, three errors to abort a given scan.
- T&W method stage 1: up 4.5 dB, down 3 dB; stage 2: up 1.5 dB, down 1 dB
- T method stage 1: 3 dB, stage 2: 1 dB
- Constant stimuli: nine steps of -2 dB from -2 to -18 TMR, eight runs.

### Data analysis

We used QUEST (Watson & Pelli, 1983) to obtain a trial-by-trial estimate of threshold. We applied the QUEST algorithm using the built-in function in MATLAB-Psychtoolbox. On each trial, QUEST calculates a probability density function (pdf) guided by pre-set prior estimates and based on the performance of previous trials. An estimate of threshold can then be extracted from the mean of this pdf (as recommended by Pelli, 1987, and King-Smith et al., 1994) and an estimate of the Quest procedure´s *confidence* from its standard deviation. This progression of the estimates across trials allowed a comparative exploration on potential time-efficiency of the adaptive methods tested based on a post hoc stopping rule. By default, the number of trials presented on each of the methods depended on a stopping rule based on the number of scans (eight) and the trials within each scan for the AS method; the number of reversals in the case of T&W and T (six second-stage reversals, see Methods). These parameters were chosen so that the number of trials and time in task were roughly equivalent across methods. These parameters are the same ones used in previous publications of our group using the T&W method (Diedesch et al., 2021; Gallun et al., 2022; Lelo de Larrea-Mancera et al., 2020, 2022; Srinivasan, 2020). In the case of the CS method, a pre-defined number of trials (16) per stimulus level (nine) that should be sufficient to calculate a psychometric function was chosen.

#### QUEST threshold calculation

QUEST was used to calculate thresholds and the slope of the psychometric function. The rationale for including QUEST was to have a single procedure that would allow for comparison across the different methods evaluated. Otherwise, there would be an issue with the fact that the different adaptive methods are targeting slightly different points in the psychometric function. Correlations with more typical procedures to estimate threshold relative to each method (e.g., averaging across the last few reversals) are provided in the OSM. QUEST was initialized with the fixed parameters of guess rate (0.5), lapse rate (set at .077), and a target threshold at 80% correct. To generate the prior distributions, a starting guess of 4 ms was used for the GIN task to have an initial guess estimate based loosely on Grose et al. (1989), Florentine et al. (1999), Samelli and Schochat (2008), Shen (2013), and Hoover et al. (2015). The TIN task was set with a starting guess of -10 TMR dB for the TIN task based loosely on Wilson et al. (2003) and Hoover et al. (2019). Ten standard deviations were allowed for each task. On each trial the QUEST procedure was set up to calculate the threshold from the mean of the posterior probability density function and the psychometric function was recalculated. The final threshold estimate is simply the mean of the pdf of the last trial that is influenced by all previous trial information. First, the CS method was analyzed allowing the parameter beta – which corresponds to the slope of the psychometric function– to vary as a free parameter from which a beta value was extracted for each participant on each task. These beta values were then used as fixed parameters to calculate the threshold separately on each of the three adaptive methods tested (AS, T&W, and T).

#### Precision

Precision was estimated as the inverse of three different types of variance: (1) *confidence,* defined as the std of the pdf used to calculate threshold by QUEST (Watson & Pelli, 1983), (2) *distance,* defined as the mean square error between a given trial threshold estimate and the final threshold estimate (considering all preset trials), and (3) *reliability*, defined as the test-retest variability of estimated thresholds. The first two are derived from the trial-by-trial information outputted by QUEST. The metric of confidence gives an estimate of how well QUEST was able to determine the threshold of the current trial based on the performance of all previous trials. Distance is the difference between the current and the final trial estimate of threshold (within-subjects mean-square-error). The metric of distance can only be calculated post hoc and is an indication of the speed at which a method converges to a threshold estimate at the end of the track. A third estimate of precision is test-retest reliability and can be estimated from the agreement between threshold estimates across two sessions. Reliability indicates how much variation may we expect to occur with repeated measurements. In sum, the precision metrics of confidence, distance, and reliability are used here to evaluate how the different adaptive methods compare to each other. Importantly, the precision metrics are used to propose shortened tracks that maintain an optimal relation to trial number by maximizing confidence and reliability and minimizing distance in the least trials possible.

#### Time-efficiency

Time-efficiency is inherently a trade-off between trial number and per-trial variance reduction. Time-efficiency in the context of this study was estimated as the number of trials necessary to obtain a threshold estimate. To explore and evaluate the potential time-efficiency of the AS method, we analyzed the precision metrics explained above. This was accomplished by estimating the average number of scans (in the case of AS) necessary to ensure a time-efficiency improvement within tolerances defined in terms of the precision metrics. This estimate (number of scans) was used to implement a post hoc stopping rule based on number of scans at the individual level. Threshold estimates obtained using the shortened tracks were then compared both within and between methods.

### Statistical analysis

To validate the AS method, we calculated test-retest correlations within method (across session) and correlations between methods using the second session (AS paired with the rest). These correlation analyses address whether the different methods yield comparable results. Correlations were compared directly across methods using a Fisher’s z-test as described in Meng et al., (1992), correcting family-wise comparisons using the Bonferroni correction. Because correlations are not the best indications of reliability in data with comparable within- and between-subject variation as we might expect with young listeners without hearing complaints performing these tasks, in complement to correlations, we compare the estimated thresholds in terms of Bland and Altman’s (1999) limits of agreement (LoA). This analysis compares the mean of two measures (plotted on the x-axis) against their difference (plotted on the y-axis) and calculates the parametric limits where 95% of the differences are expected to occur (95% LoA). This calculation is taken from the mean differences across measures ± 1.96 times their standard deviation. The mean difference itself represents a measure of bias that indicates systematic differences favoring one measure or the other. As with the correlations, we repeat this analysis for both within-method (test-retest) and between-method pairs (using the second session).

For the time-efficiency exploration, a visual inspection of the average trial-by-trial estimates of precision considered was carried out to determine the scan number where the precision estimates held an optimal trade-off with number of trials. A shortened AS version was then compared to the rest of the adaptive methods. For each task, a repeated measures 4 × 2 ANOVA was applied to the threshold estimates of the adaptive methods, and a shortened version of the AS method. The within-subject factors Method (four levels: AS-short, AS, T&W, T), and Time (two levels: first, second) were used. The main factor effect of Method was considered to evaluate equivalence in estimated thresholds across the tested adaptive methods. The interaction between Method*Time was considered to reveal differences across method in the differences across session (described and analyzed in the validation section as bias in the LoA analysis). Pertinent post hoc comparisons are conducted correcting p-values for multiple comparisons using the Bonferroni-Holm method.

## Results

Results are divided in two sections for clarity: (1) validation and (2) time-efficiency exploration. The first section focusses on validating the AS and addresses the extent to which the AS method tracks thresholds with similar precision compared to contemporary methods. The second section focusses on time-efficiency and explores the relationship between a shortened AS method and precision.

### Validation of the AS method

#### Is the AS a valid method to track psychometric thresholds?

**Fig. 2:**
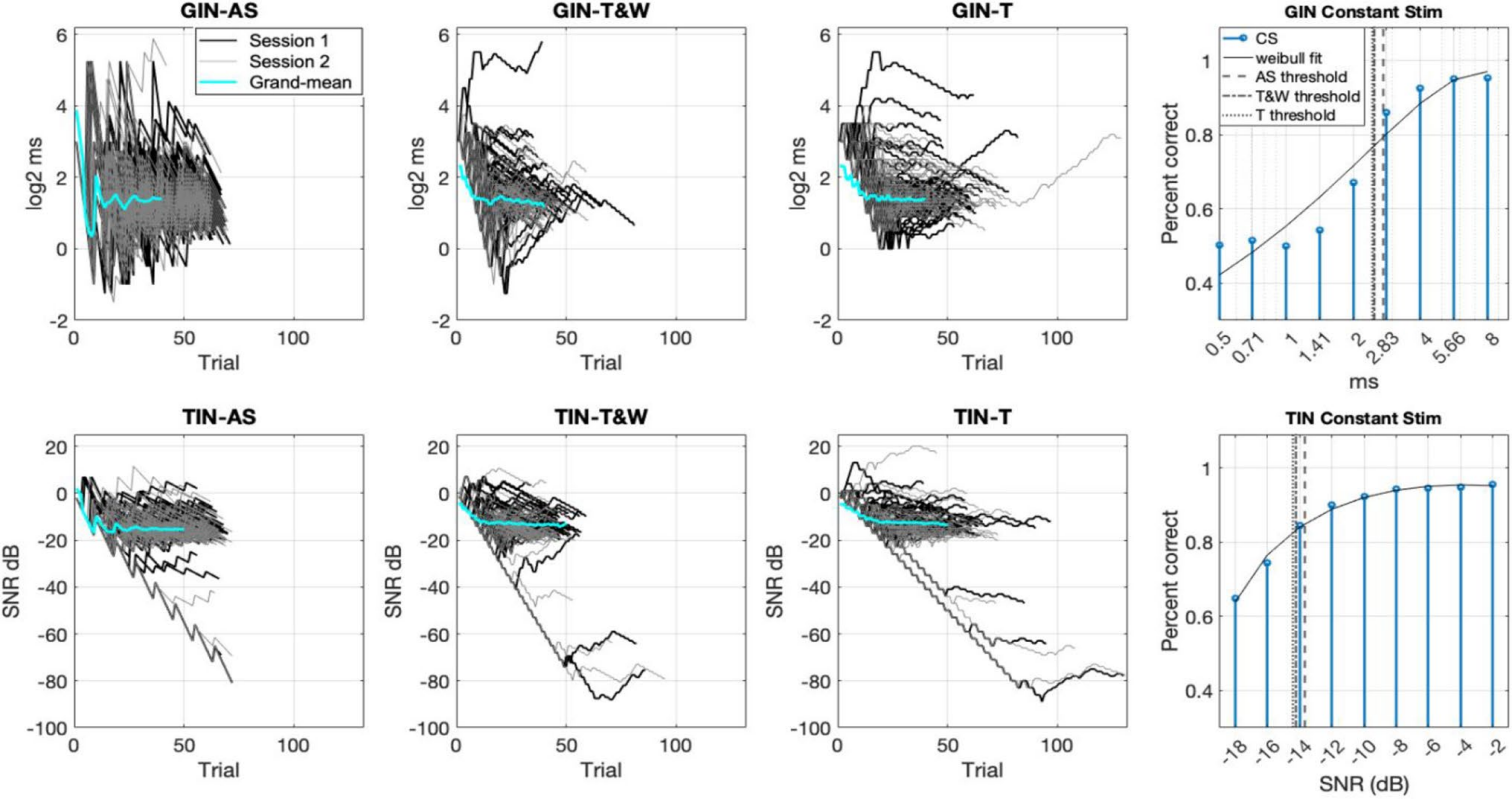
The stimulus level delivered per trial on average (±SEM) for the adaptive methods across two sessions and the average threshold obtained with the constant stimulus method in a dashed line. The gap-in-noise (GIN) task is shown on the top and the tone in noise (TIN) task in the panels on the bottom. Panels on the far-right show percent correct per stimulus level in the constant stimulus (CS) method and the threshold obtained with the adaptive methods in vertical lines (adaptive scan (AS) dashed red; transformed and weighted up-down method (T&W) solid green; transformed up-down method (T) dashed blue)

We will address this question by first comparing test-retest reliability across the adaptive methods, and then analyzing the extent to which different methods track similar threshold estimates per participant. Before we show formal statistical testing to validate the AS method, we first present the raw data as an overview of the study and results. Figure 2 describes the stimulus levels being delivered across trials for each participant on the different sessions performed on each method and each task. The purpose of this figure is to explore visually the overall relationship between the tested methods. The panels on the far right depict the percentage correct achieved on average on the different stimulus values chosen for the method of CS. The thresholds for the adaptive methods are depicted in the CS psychometric function as vertical lines. The panels on the left depict all the adaptive runs of each adaptive method, and the between-subject mean averaged across sessions (grand-mean) (Fig. 2; panels on the left). As can be seen in Fig. 2, the different adaptive methods are adapting to similar parametric regions in both sessions.

#### What is the reliability of the threshold estimates within each method?

**Fig. 3:**
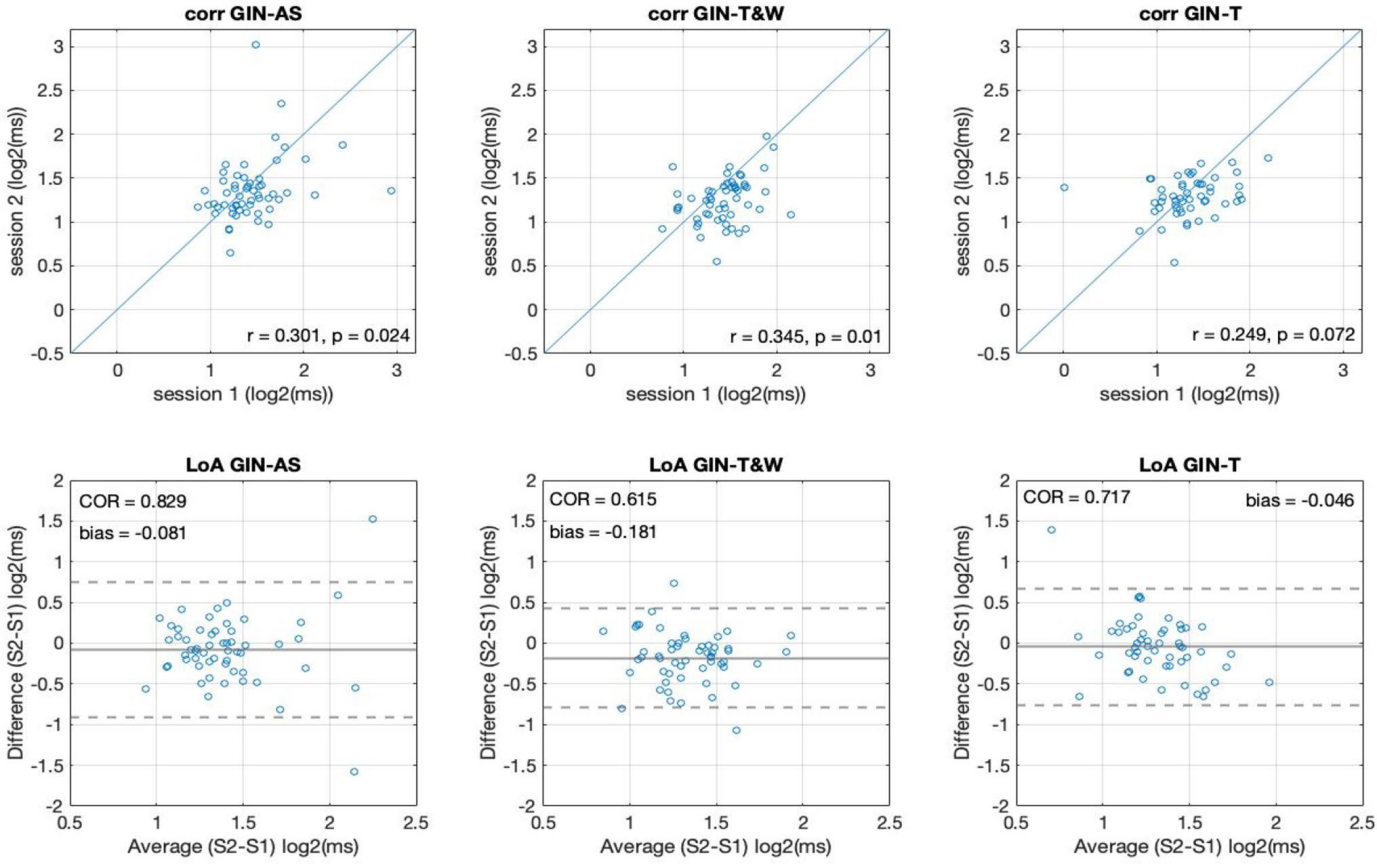
The correlations (**top**) and limits of agreement (LoA; **bottom**) between sessions as indications of reliability in all adaptive methods of the gap-in-noise (GIN) test

**Fig. 4:**
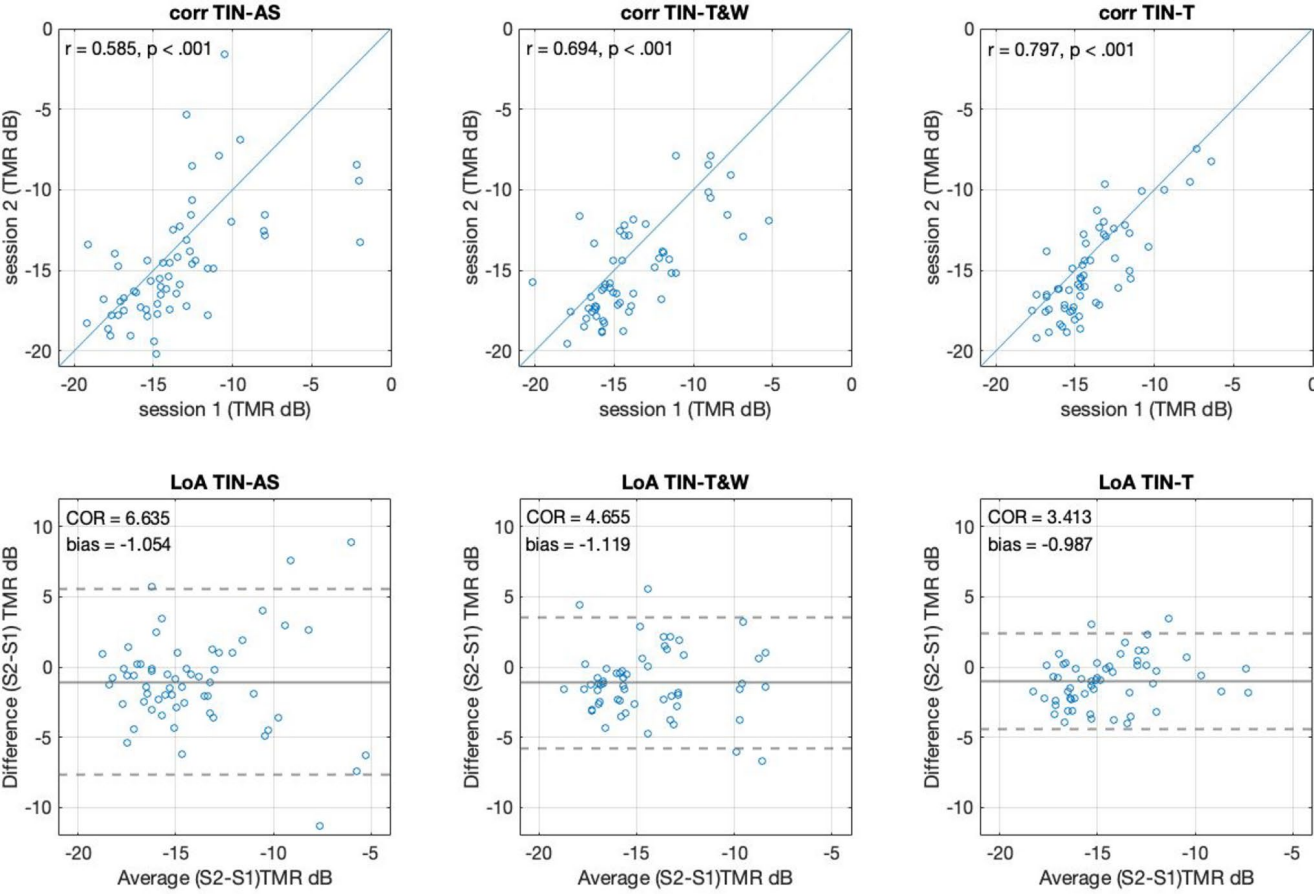
The correlations (**top**) and limits of agreement (**bottom**) between sessions as indications of reliability in all adaptive methods of the tone-in-noise (TIN) test

We first addressed the precision of repeated measures (test-retest reliability) within each of the adaptive methods. Correlations and limits of agreement between testing sessions for the GIN task (Fig. 3) and TIN task (Fig. 4) show the reliability for each measure. In the case of the GIN task, correlations were *r* = .31, *p =* .02 for the AS method, *r* = .34, *p =* .01 for T&W, and *r* = .24, *p =* .07 for the T method. These between-session correlations do not differ between-methods in a statistically significant way according to Fisher’s r to z test (*z*s < 0.53, *p*s = .99). This indicates the AS method has similar reliability to other established adaptive methods as indicated by correlations in the GIN task (Fig. 3 upper panels). However, correlations might not be the best measure of reliability in some conditions (Bland & Altman, 1999), so we also conducted a LoA analysis (Fig. 3 lower panels). Here we observe the coefficient of reliability (COR) – which gives magnitude of expected error (95%) between repeated measures – was of .82 log2(ms) or 1.76 ms for the AS method, .61 log2(ms) or 1.52 ms for the T&W method, and .71 log2(ms) or 1.63 ms for the T method. These are the magnitudes in which we may expect variation across repeated measures. Further, we observed a slight bias favoring performance in Session 2 by .081 log2(ms) or 1.05 ms for the AS method, .18 log2(ms) or 1.1 ms for the T&W method, and .046 log2(ms) or 1.03 ms for the T method. This indicates a systematic improvement of around 1 ms, perhaps involving task learning after the first session, across all adaptive methods. In sum, all methods show similar reliabilities in the case of the GIN task.

In the case of the TIN task, correlations were *r* = .58*, p <* .001 for the AS method, *r* = .69*, p <* .001 for T&W, and *r* = .79*, p <* .001 for the T method. These correlations between testing sessions do not differ in a statistically significant way according to Fisher’s r to z test (*z*s < 0.97, *p*s > .05). This indicates the AS method has similar reliability to other established adaptive methods as also indicated by correlations for the TIN task (Fig. 3 upper panels). Moreover, the LoA analysis indicated the coefficients of reliability (COR), indicating the range where we might expect 95% of differences between measures to occur, were of 6.63 dB for the AS method, 4.65 dB for the T&W method, and 3.41 dB for the T method. Again, we may observe a small bias favoring performance in Session 2 by 1.05 dB for the AS method, 1.1 dB for the T&W method, and 0.98 dB for the T method (Fig. 3 lower panels). In sum, the reliability observed by correlations was similar across the tested adaptive methods. In the case of the LoA analysis, the bias was similar across methods (about 1 dB), but the COR was 1.98 dB wider in the case of the AS in comparison to the T&W method and 3.22 dB wider when compared to the T method.

#### Do the different methods converge on similar estimates?

**Fig. 5:**
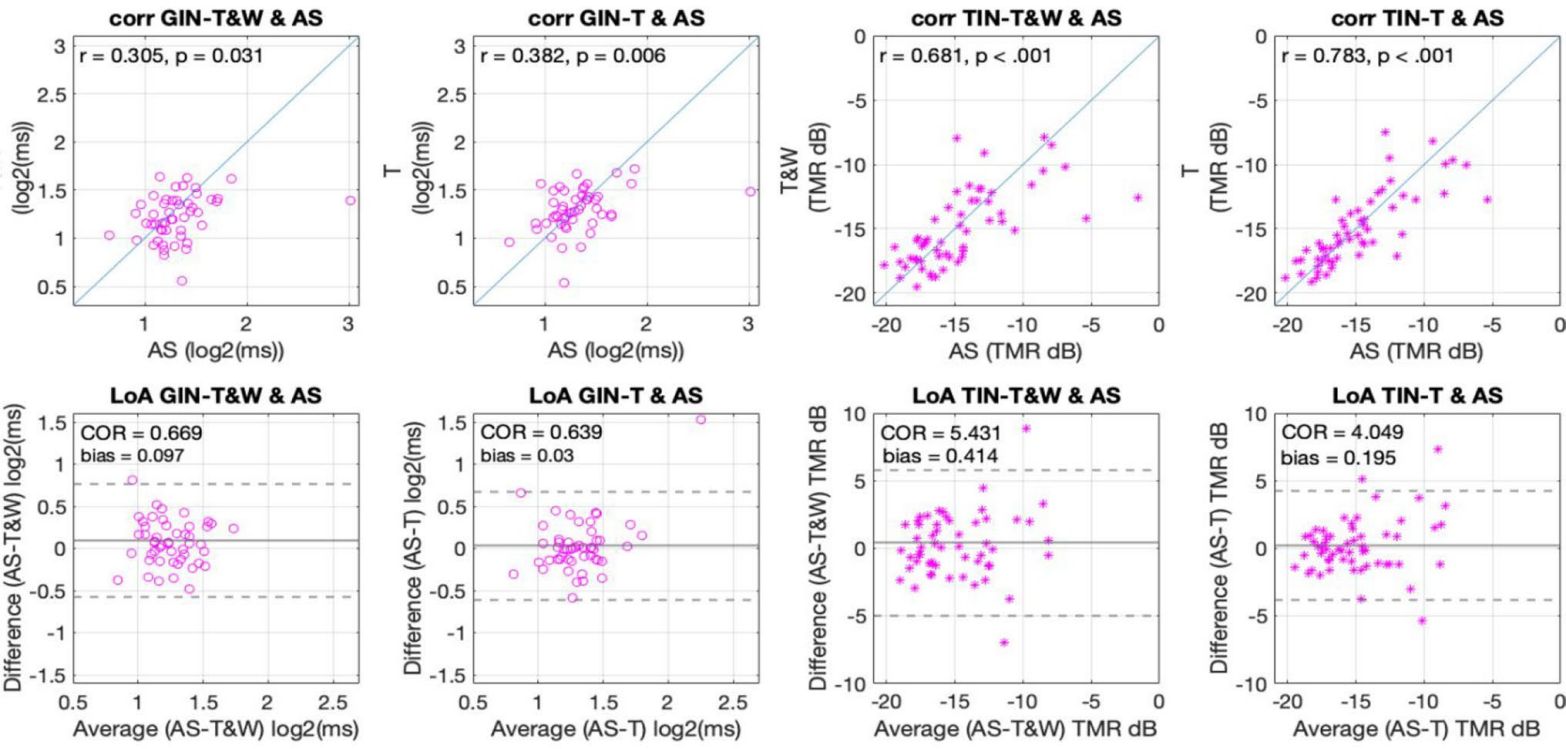
The correlations (**top**) and limits of agreement (LoA; **bottom**) between methods on the second session of testing as indicates of equivalence across adaptive methods of both the gap-in-noise (GIN; left four panels) and the tone-in-noise (TIN; right four panels) tests

The second question in the validation section addresses whether the different adaptive psychophysical methods that have been previously validated (T&W and T) converge on similar measurements to the AS method. To this end, we performed correlations of the AS method and each of the other adaptive methods in the second session where practice effects might be less influential. After this, we compared the obtained correlations to the ones assessing repeatability shown and reported above. Figure 5 shows the scatterplots and LoA analysis relating threshold estimation of the AS and other adaptive methods used.

For the GIN task, correlations were of *r* = .305*, p =* .03 for AS and the T&W method, and *r* = .38*, p =* .006 for AS and the T method (Fig. 5 top left panels). Importantly, these correlations were not statistically different to the test-retest correlations according to Fisher’s *r-to-z* test (*z*s < 0.83, *p*s > .99). This means the difference between methods in terms of the estimated threshold does not exceed the error associated with repeated measurement. Moreover, in the LoA analysis we observed a COR between AS and T&W of .66 log2(ms) or 1.58 ms, and between the AS method and the T method of .63 log2(ms) or 1.54 ms, both of which are within the range of the test-retest coefficients. In terms of bias, the AS method estimated ≤ .09 log2(ms) or 1.06 ms worse thresholds than the other adaptive methods tested; this again is very close to the bias associated with within-method test-retest measures.

For the TIN task, correlations were *r* = .68, *p <* .001 for AS and the T&W method, and *r* = .78, *p <* .001 for AS and the T method. Importantly, these correlations were not statistically different to the test-retest correlations according to Fisher’s *r-to-z* test (*z*s < 2.01, *p*s > .13). This means the between-method difference does not exceed the within-method repeated-measures error. In the LoA analysis, we observed the COR between AS and T&W was of 5.43 dB and between the AS method and the T method was of 4.04 dB, both of which are similar to the test-retest coefficients. In terms of bias, we observed worse thresholds calculated by the AS method by less than 1 dB.

### The quest for time-efficiency

#### Time-efficiency exploration

**Fig. 6:**
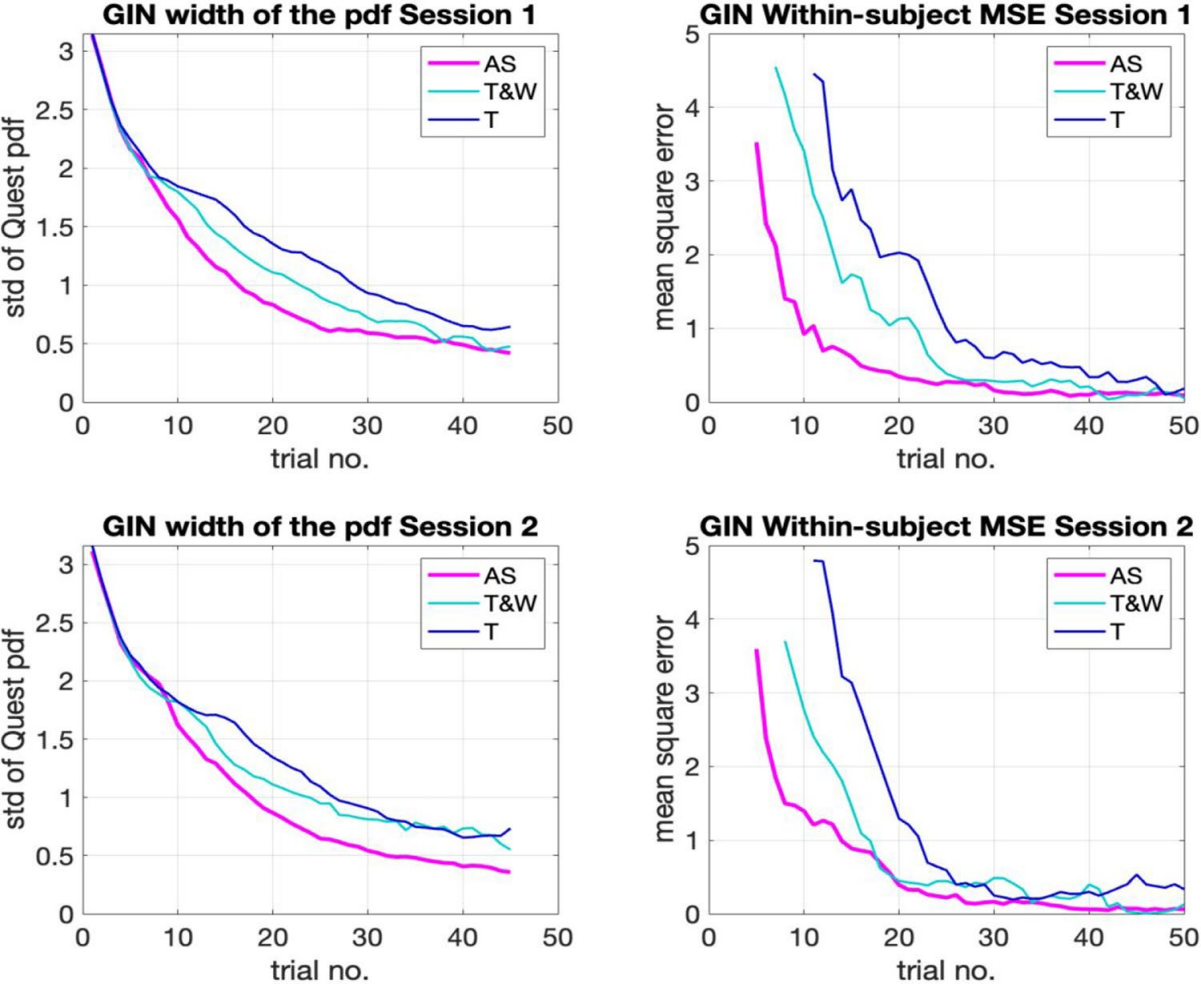
The average by-trial variance estimates of confidence (**left**) and distance (**right**) of each of the adaptive methods in the gap-in-noise (GIN) test

**Fig. 7:**
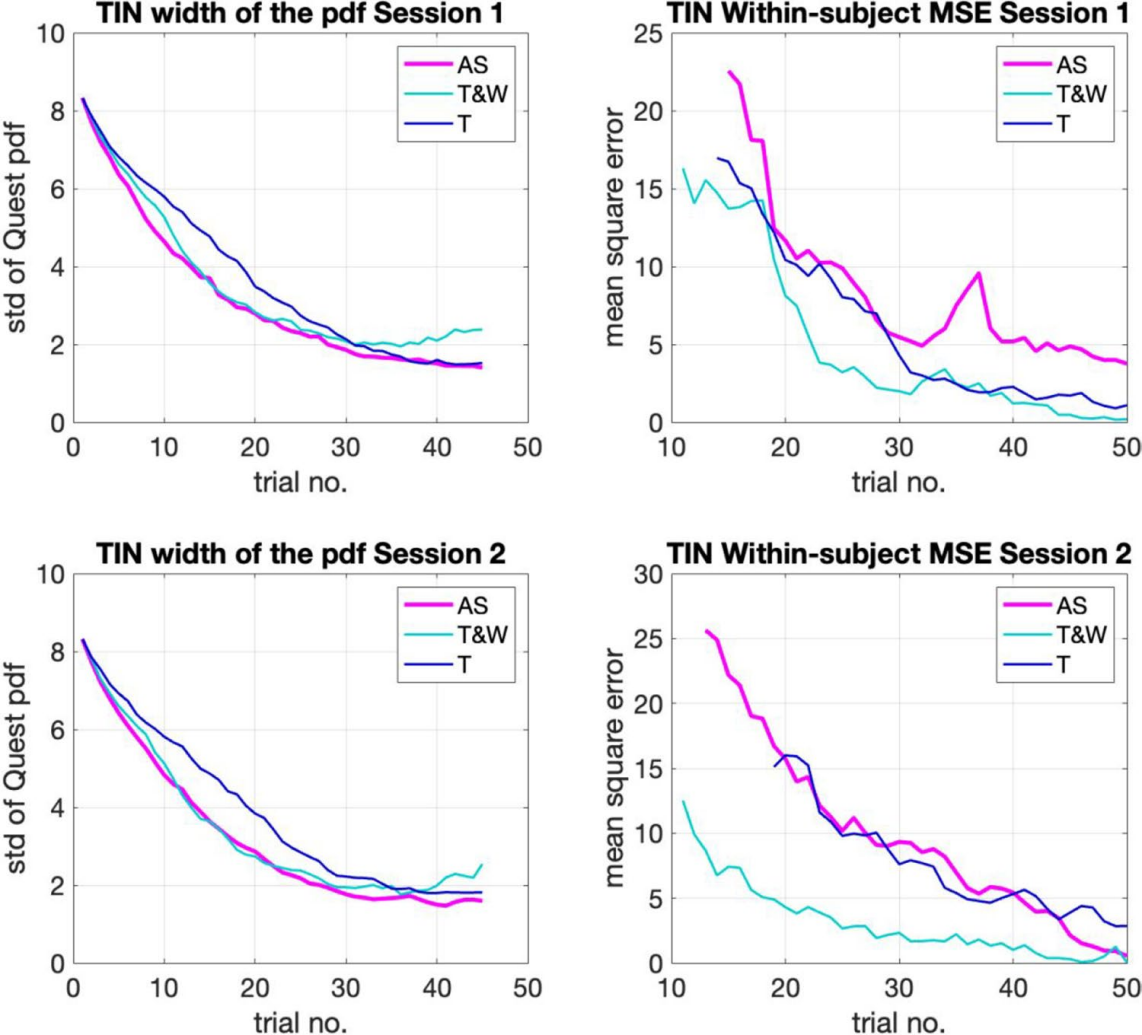
The average by-trial variance estimates of confidence (**left**) and distance (**right**) of each of the adaptive methods in the tone-in-noise (TIN) test

To conduct this exploration, we analyzed the relationship between trial number and the two kinds of measurement variance that remain unexplored so far: (1) confidence, understood as the width of the likelihood function used by the QUEST procedure (std of the pdf); and (2) distance, understood as the within-subject MSE relative to the final threshold estimate. Figures 6 and 7 show these estimates for each task separately considering session (top vs. bottom), metric (right confidence, left distance), and adaptive method (color coded). After visual inspection of the trial-by-trial precision metrics, we determined the adaptive tracks could be shortened considerably and still get a similar threshold calculation.

As we may observe in Figs. 6 and 7, the measures of variance reduce as trial number increases in all methods. In other words, threshold calculation grows in precision as it is informed by more trials. This reduction seems to continue until the end of the test in both cases (GIN and TIN). All methods display similar reductions on their trial-by-trial average variance (increase in precision) across both metrics. The AS seems to reduce its variance estimates faster than the T and T&W methods in the GIN task (Fig. 6). The T&W seems to have an advantage in the distance metric of the TIN task.

#### Time-efficiency comparison

**Fig. 8:**
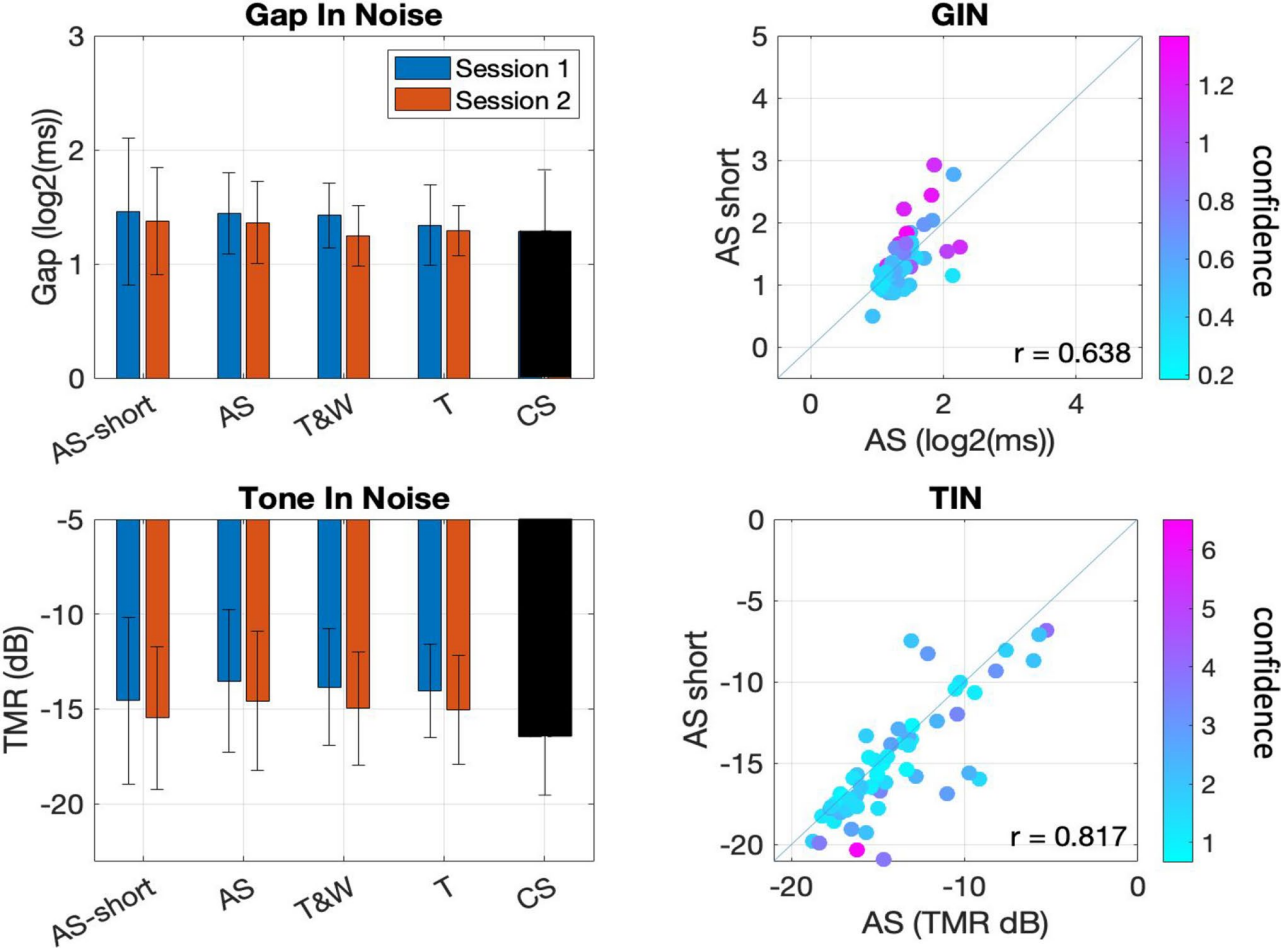
The left panels show the average threshold calculated for each method including the shortened version of the adaptive scan (AS) method (AS-27). Lower values indicate better performance, error-bars indicate standard deviation. Panels on the right show the correlation between the threshold calculated from the full tracks with eight scans on the AS in the x-axis and the shortened version of the AS method on the y-axis. The graded color code indicates the confidence of threshold calculation (SD of PDF). The top row represents the gap-in-noise (GIN) task and the bottom, the tone-in-noise (TIN) task

After the visual inspection of the average precision change across trials reported above, we estimated that three scans (of nine trials) would be a reasonable stopping rule to maximize precision in the least number of trials for the adaptive scan. The rate of change in the precision metrics is less dramatic after trial 27. Figure 8 shows a comparison of the shortened version of the AS scan that was calculated from the last trial of the third scan (trial 27 in a track with no interrupted scans) *as if* the track had ended there. Panels on the left afford visualization in terms of mean threshold comparison to the full version of the AS, and the other adaptive methods. The CS method is also displayed for descriptive purposes, but only adaptive algorithms are statistically compared in this section. Panels on the right of Fig. 8 show the correlation between the AS thresholds reported above and the post hoc shortened version. The color code indicates the precision metric of confidence (higher values, less confidence).

To test the potential differences between the shortened version of the AS method and the rest of the adaptive methods (full AS, T&W, T) across two sessions, we conducted repeated-measures 4 × 2 ANOVAs for each task. According to the software G*Power 3.1 (Faul et al., 2007), each of these repeated-measures tests had a statistical power of 99% to find a medium effect size (*f =* .25) and 97% to find a small effect size (*f =* .15) with the tested sample (*n =* 70) across eight repeated measures. For the GIN task, there was no significant effect of Method (*f =* 0.91, *p* = .43), and no significant interaction across sessions (Method*Time, *f =* 2.25, *p* = .08).

In the case of the TIN task, there was a statistically significant effect of Method (*f =* 4.95, *p* = .003, *η*^*2*^ = .033). Post hoc pairwise comparisons revealed the significant differences were under 1 dB in magnitude and occurred only in pairs that included the AS-short method (AS, *t =* 3.58, *p =* .003, *mean difference* = 0.88 dB; T&W, *t =* 2.58, *p =* .043, *mean difference* = 0.64 dB; T, , *t =* 2.93, *p =* .019, *mean difference* = 0.72 dB). All other pairwise comparisons had corrected p values > .95. There was no significant interaction of the factor Method across sessions (Method*Time, *f =* 0.17, *p* = .91).

**Fig. 9:**
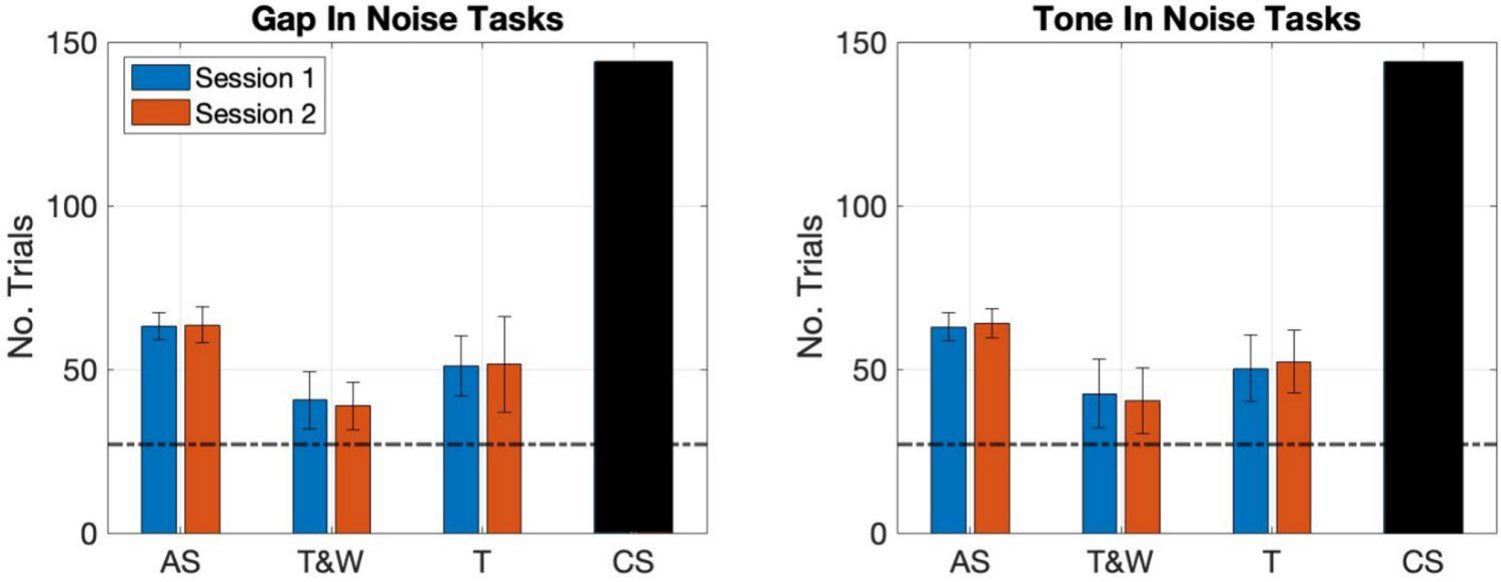
The number of trials required on average to finish a run of each of the adaptive methods tested across two runs. The dotted line at trial 27 indicates the maximum possible length of the proposed adaptive scan (AS) short tracks. The panel on the left represents the gap-in-noise (GIN) task and panel on the right the tone-in-noise (TIN) task. In the case of the constant stimulus (CS) method, both blocks of 72 trials were delivered on the third day of the experiment in a single session as described in the procedures and only one threshold is extracted from 144 trials

In sum, the trial-by-trial analysis was able to guide our decision of when we could optimize the number of scans in AS while losing little precision in the average performer. The group-level differences found between the shortened-AS method and the rest of the adaptive methods tested were restricted to the TIN task and were of less than 1 dB. Lastly, Fig. 9 shows the average length of the tracks presented with each method as a summary of the quest to reach time-efficiency. In the figure it may be seen that every adaptive method chosen represents an increase in efficiency relative to the CS method of about half the trials necessary to achieve a threshold estimate. Further, the trial number 27 is marked with a dotted line (Fig. 9) to indicate the relative increase in efficiency with the AS short version relative to the rest of the methods tested.

## Discussion

In this work we introduced an AS method and validated it against known adaptive up-down methods (T, T&W), and the method of constant stimulus (CS). To do this, we had 70 undergraduate students complete two different tasks (GIN and TIN), each of which was using each of the four methods in a fully crossed within-subject design. We observed similar adaptation across the parametric space as well as similar estimated thresholds across all adaptive methods tested in both tasks (see Fig. 2). Further, the test-retest reliability was also comparable across all methods for both tasks (Figs. 3 and 4). Lastly, the correlations between the thresholds of the different adaptive methods (Fig. 5), were not different to the within-method (test-retest) correlations indicating similar behavior in all methods. Overall, these observations converge in the validation of the AS as an adaptive method for psychophysical testing.

The focus to investigate more time-efficient methods for auditory assessment is already addressed successfully in the validation of AS because of its inherent characteristics in relation to embedded practice trials. The AS method, by integrating easy trials into its adaptive regions by design addresses some of the challenges in time-efficiency including quantifying these trials and allowing people to keep track of the cue they are listening to. The observation that the AS method adapts to a similar parametric region on a similar time scale to the other methods already indicates a benefit of using the AS. However, it took considerably more trials to achieve the AS threshold compared to the other adaptive methods tested with the preset parameters (e.g., eight scans; see Fig. 9). To address the potential improvement in terms of number of trials presented while maximizing the method’s precision, we calculated the trial-by-trial progression of measurement variance (Figs. 6 and 7) and determined a time-efficient shortened-version of the AS with only three scans and a maximum of 27 trials. This short-AS was not different in terms of estimated threshold than the full version or the other adaptive methods, and the test-retest reliability also did not differ (see Fig. 8). This is taken as evidence that the short-AS could be used to reach a threshold estimate for the average participant. We note that this estimate is coarser and does not address some cases of performance. For example, a few participants were able to do extremely well in the TIN task. The different adaptive methods were able to track this, but it took them longer to converge. The AS short would only be able to tell this performance is better than average but not by how much. A screening scenario where the goal is to distinguish between normal and impaired performance is more adequate for the type of difficulties the AS short could face in reaching a good estimate.

We did not explore different stopping rules that could be implemented in the other adaptive methods to achieve similar shortened versions, nor did we compare their potential in terms of time/precision trade-off. Although interesting, it is beyond the scope of the current work. Future work could propose different stopping rules for the more traditional up-down methods and compare their relative efficiency. Here we are satisfied with showing an adaptive method that can adapt on a range rather than a single value and achieve similar threshold estimates to the methods we have been using (T&W) or that are typical (T) even with a shortened-version that represents an improvement in the way it integrates trials that are easier than threshold (practice) and the number of trials needed to achieve a threshold. Also, the AS design is such that some of the benefits of adaptive and fixed staircases are combined to avoid some of their problems. The adaptive benefits include the ability to deliver stimuli at more diverse levels quickly, that is, jump to new stimulus levels rather than being stuck in a potentially inadequate parametric region as in fixed staircases; the fixed benefits involve no requirement of an a priori parametric model of the listener's ability to perform the task, which is seldom the case with the general public nor in clinical populations. The next steps are to test the algorithm with other hearing tests, and with different populations with different hearing sensitivities. A promising future direction is to use the shortened AS with the general population to screen for hearing difficulties in a range of different auditory processing assessments.

We note that other methods could have been included in this comparison like the classic *up-down* method first introduced by Dixon and Mood (1948), the *weighted up-down* method described by Kaernbach (1991), or parametric methods such as the stimulus selection algorithm used in QUEST (Watson & Pelli, 1983). Additionally, the list of parameters that can be manipulated orthogonally in experimental conditions that we did not include is extensive as it comprises: up-down rules, step-sizes, their size ratios, the initial value, the minimum and maximum possible values, and the inclusion or exclusion of different staircase stages. Lastly the different psychophysical assessments that could have been used and where we expect some variation to occur also goes beyond the selected two. However, it is our perspective that the current piece serves to introduce and validate the AS method, as well as suggests a stopping rule of three scans for a maximum of 27 trials that may serve well in scenarios where a small amount of lost precision is admissible, such as in screening procedures where coarse discrimination is of higher value. We further note that more analysis could have been performed on this data such as searching for inflection points in the psychometric functions and analyzing those instead of the 80% threshold, searching for the calculated slopes of the psychometric function and comparing those, or we could have performed different types of simulations to test the different methods even further. However, again, it is our perspective that the analysis provided here is sufficient to introduce and validate the AS method as well as suggest a shortened version for exploration and further study.

In conclusion, the work presented here is a step towards introducing more time-efficient testing of listening abilities that may compliment clinical practice to the extent that they are able to circumvent the costs associated to them. Future work is needed to demonstrate the usability and utility of these portable automated rapid tests.

### Supplementary Information

Below is the link to the electronic supplementary material.

Supplementary file1 (DOCX 524 KB)

## Data Availability

None of our experiments was pre-registered. All materials of the study including stimuli, tasks are available online for free (see https://ucrbraingamecenter.github.io/PART_Utilities/).

## Data Availability

All data and analysis code are available upon request: Please contact e.lelodelarrea@northeastern.edu.
