# Supplementary material for: Validation of the adaptive scan method in the quest for time-efficient methods of testing auditory processes
Source: Atten Percept Psychophys. 2023 Jun 22;85(8):2797–810. doi: 10.3758/s13414-023-02743-z (PMC10600050; doi:10.3758/s13414-023-02743-z)
Supplement: Supplementary file 1 — Supplementary file1 (DOCX 524 KB) [file 13414_2023_2743_MOESM1_ESM.docx]

Supplemental Materials.

One challenge in comparing these different methods for psychophysical testing is that they constitute non-parametric approaches to different threshold targets and the way the thresholds are calculated in each case might not be equivalent. To address this issue we used by QUEST (Watson & Pelli, 1983) which uses a maximum-likelihood estimation of a pre-set threshold level based on the history of performance and a prior probability distribution. The advantage of this procedure is that it can be applied across all the methods mentioned here. Although procedures like QUEST are often used to guide stimulus selection (e.g. Shen, 2013) here we use it to have an equivalent means of estimating threshold across different methods of stimulus delivery. Correlations with more typical procedures to estimate threshold relative to each method (e.g. averaging across the last few reversals) are provided here for a demonstration of the validity of the QUEST approach across the diverse methods used. In this study, we use QUEST to conduct comparisons across methods in terms of precision and time-efficiency.

## Validation of the QUEST procedure

In this section QUEST is validated in the context of the full dataset (no outlier rejection). As can be observed in figures 3 and 4 QUEST is strongly associated to the more typical and previously used procedures to extract threshold. Datapoints that deviate from midline also correspond to wider distributions of the posterior probability density functions. These individual datapoints are analyzed further in the supplemental materials to better understand the cases in which one method or another might be more suitable.

**Figure S1.** Shows the correlations between the typical procedures for threshold estimation and QUEST for each method in each session of testing in the GIN task. The color-scale indicates the within-subject error of the estimate in QUEST based on the standard deviation of the posterior pdf from which threshold is taken as the mean.

**Figure S2.** Shows the correlations between the typical procedures for threshold estimation and QUEST for each method in each session of testing in the TIN task. The color-scale indicates the within-subject error of the estimate in QUEST based on the standard deviation of the posterior pdf from which threshold is taken as the mean.
